# New Candidates for Biomarkers and Drug Targets of Ischemic Stroke—A First Dynamic LC-MS Human Serum Proteomic Study

**DOI:** 10.3390/jcm11020339

**Published:** 2022-01-11

**Authors:** Aleksandra Turek-Jakubowska, Janusz Dębski, Maciej Jakubowski, Ewa Szahidewicz-Krupska, Jakub Gawryś, Karolina Gawryś, Agnieszka Janus, Małgorzata Trocha, Adrian Doroszko

**Affiliations:** 1 Department of Neurology, 4th Military Hospital, Weigla 5, 50-556 Wroclaw, Poland; lek.aturek@gmail.com (A.T.-J.); karolina.maria.gawrys@gmail.com (K.G.); 2 Institute of Biochemistry and Biophysics, Polish Academy of Sciences, Pawińskiego 5a, 02-106 Warszawa, Poland; jasio.ibb@gmail.com; 3 Lower Silesian Centre for Lung Diseases, Grabiszyńska 105, 53-439 Wroclaw, Poland; maciek.jak@gmail.com; 4 Department of Internal Medicine, Hypertension and Clinical Oncology, Wroclaw Medical University, Borowska 213, 50-556 Wroclaw, Poland; ewa.szahidewicz-krupska@umed.wroc.pl (E.S.-K.); jakub.gawrys@umed.wroc.pl (J.G.); jaga116@poczta.onet.pl (A.J.); 5 Department of Pharmacology, Faculty of Medicine, Wroclaw Medical University, Mikulicz-Radecki 2, 50-349 Wroclaw, Poland; malgorzata.trocha@umw.edu.pl

**Keywords:** ischemic stroke, biomarker, therapeutic target, proteomics, liquid chromatography–mass spectrometry (LC-MS)

## Abstract

(1) Background: The aim of this dynamic-LC/MS-human-serum-proteomic-study was to identify potential proteins-candidates for biomarkers of acute ischemic stroke, their changes during acute phase of stroke and to define potential novel drug-targets. (2) Methods: A total of 32 patients (29–80 years) with acute ischemic stroke were enrolled to the study. The control group constituted 29 demographically-matched volunteers. Subjects with stroke presented clinical symptoms lasting no longer than 24 h, confirmed by neurological-examination and/or new cerebral ischemia visualized in the CT scans (computed tomography). The analysis of plasma proteome was performed using LC-MS (liquid chromatography–mass spectrometry). (3) Results: Ten proteins with significantly different serum concentrations between groups volunteers were: complement-factor-B, apolipoprotein-A-I, fibronectin, alpha-2-HS-glycoprotein, alpha-1B-glycoprotein, heat-shock-cognate-71kDa protein/heat-shock-related-70kDa-protein-2, thymidine phosphorylase-2, cytoplasmic-tryptophan-tRNA-ligase, ficolin-2, beta-Ala-His-dipeptidase. (4) Conclusions: This is the first dynamic LC-MS study performed on a clinical model which differentiates serum proteome of patients in acute phase of ischemic stroke in time series and compares to control group. Listed proteins should be considered as risk factors, markers of ischemic stroke or potential therapeutic targets. Further clinical validation might define their exact role in differential diagnostics, monitoring the course of the ischemic stroke or specifying them as novel drug targets.

## 1. Introduction

Stroke is among the most common causes of death and permanent disability in adults [1]. Eighty percent of strokes are caused by occlusion of the vessel lumen by thrombotic or embolic material [2]. Animal studies have shown that at the time of stroke the blood-brain barrier (BBB) is damaged and becomes permeable for greater amounts of proteins [3]. This mechanism may also occur in humans [4]. Some reports suggest that the measurement of brain-derived proteins in plasma could be useful in diagnosis and monitoring acute phase of ischemic stroke [5]. Furthermore, serum stroke biomarkers might be useful in risk stratification to determine the optimal way of treatment for specific patients.

To date, there are several defined potential biomarkers linked to the stroke. They demonstrate some prognostic value regarding long-term disability [6,7]. Recent studies point at the possible involvement of osteoprotegerin, serum free hemoglobin, S-100 protein, brain natriuretic peptide (BNP) as stroke biomarkers, but none of them are characterized by a sufficient sensitivity and specificity to be recommended in clinical practice [8,9,10,11]. Others, such as D-dimer, lipoprotein-associated phospholipase A2 (Lp-PLA2), fibrinogen, myelin basic protein, neurospecific enolase (NSE) glial fibrillary acidic protein, heart-type fatty acid binding protein (H-FABP), apolipoprotein C I and III (ApoC I and III), von Willebrand factor (vWF), matrix metalloproteinase-9 (MMP-9), monocyte chemotactic protein-1 (MCP-1) and highly sensitive C-reactive protein (hsCRP), are mostly related to risk factors of ischemic stroke and are useless in the early diagnosis [12,13,14].

Stroke is increasingly understood as “acute cerebral syndrome,” which emphasizes the analogy to acute coronary syndrome and the classic risk factors for stroke include similarly the hypertension, diabetes and atrial fibrillation. Its primary and secondary prevention is currently based on the atherosclerotic plaque stabilizing management with statins (and ezetimibe when the therapeutic goal is not achieved) [15], as well as appropriate antiplatelet/antithrombotic treatment and antihypertensive treatment including angiotensin converting enzyme (ACE) inhibitors or angiotensin receptor blockers (ARBs) in combination with thiazide-like diuretics or/and with dihydropyridine-like calcium channel blockers (CCBs).

The detection of biochemical markers of vascular brain injury should have similar implications as diagnostic and prognostic value of troponins or creatine kinase myocardial band isoenzyme (CK-MB) in myocardial infarction [16]. However, there are still no defined markers of acute ischemic events in the central nervous system that would closely correspond to the extent of ischemic foci and show the dynamics of changes over time. They would also make it possible to recognize a recurrent stroke more quickly than the appearance of neuroimaging changes [17].

The aim of the study was an attempt to falsify the hypothesis regarding no significant differences in the serum proteome between patients with ischemic stroke when compared to control without acute cerebral ischemia using a Liquid Chromatography—Mass Spectrometry (LC-MS) technique. The dynamic relationships between the course of the disease and the level of identified potential candidate proteins were also analyzed.

## 2. Materials and Methods

### 2.1. Bioethics Statement, Protocol Approvals and Patient Consents

All experiments were conducted and approved in accordance with the guidelines of the local Bioethics Committee and adhered to the principles of the Declaration of Helsinki and Title 45, U.S. Code of Federal Regulations, Part 46, Protection of Human Subjects (revised 13 November 2001, effective 13 December 2001). All participants provided their written consent to participate in the study. The written consent form had been approved by the ethics committee.

### 2.2. Recruitment of Patients, Study Design and Groups Description

A total of 31 patients at age of 29–80 years with diagnosed acute ischemic stroke at the Neurology Department of the 4th Military Clinical Hospital (Wroclaw, Poland) were included into the study. The control group constituted 28 volunteers hospitalized at the Internal Medicine Department, which had similar comorbidities, cardiovascular risk factors and were demographically matched to the study group. Control patients were matched for age, gender, cardiovascular risk factors, and demographic characteristics. Exclusion criteria for both groups were: lack of medical history, anemia, thrombocytopenia, past nervous system diseases (including previous ischemic or hemorrhagic stroke), past head injuries, atrial fibrillation (including paroxysmal and requiring oral anticoagulant treatment), malignancies, chronic inflammatory diseases, current infections, chronic kidney disease (eGFR < 45 mL/min/1.73 m^2^), medications including drugs potentially affecting the obtained results (anticoagulants, anticonvulsants, contraceptives, hormone replacement therapy) and inability to provide informed consent.

**Figure 1:**
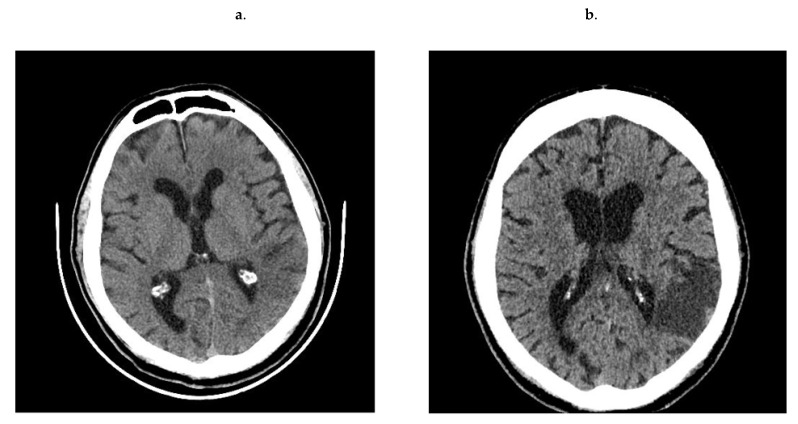
Ischemic lesion evolution between 1st day (**a**) and 7th day (**b**) in the no-contrast CT scans.

Patients positively recruited presented clinical symptoms of stroke lasting no longer than 24 h, confirmed by neurological examination and/or new cerebral ischemia visible in computed tomography scan (CT) (Figure 1). Following the diagnosis of stroke, they were additionally examined twice: on the 3rd day (to distinguish the stroke from TIA) and on the 7th day after ischemic stroke. The control subjects formed group C. Physical examination was followed by blood collection and neurological examination. In all groups, cardiovascular risk stratification was performed. Differential paired-analyses of the plasma proteome and peptidome were performed between control group and study groups—A vs. C, B vs. C and A + B vs. C.

### 2.3. Blood Collection

Blood samples for laboratory tests were taken on an empty stomach and in atraumatic conditions after single puncturing the ulnar vein once with the S-Monovette set (Sarstedt AG & Co, Sarstedt, Germany). 

Serum was obtained for creatinine, estimated glomerular filtration rate (eGFR), glucose, lipid profile, urea, uric acid, sodium, potassium and hsCRP. Whole blood collected in a tube with an activator of coagulation was centrifuged for 15 min. at 1000× *g* in 45 min from its collection. Preserved in Eppendorf, serum was transferred to accredited university hospital laboratory. Test were performed using routine methods. 

### 2.4. Preparation of Plasma for Proteomic Analysis

Blood collected in S-Monovettes (EDTA) was centrifuged for 15 min at 4 °C to obtain samples of the plasma. Then samples were stored at −80 °C until further determinations were made. After thawing, the plasma was diluted with ammonium bicarbonate containing 25% acetonitrile and the peptides were gradually separated. In order to remove proteins with high molecular weight, the diluted plasma samples were centrifuged using special filters at a temperature of +4 °C. The filtrate was centrifuged to yield a peptide fraction which was in turn dried using a vacuum concentrator. The dried peptides were dissolved in 0.1% formic acid solution and purified and then loaded onto the chromatography column using a step gradient of acetonitrile (ACN): 0–5 min-0% ACN, 5–9 min-60% ACN, 9–12 min-98% ACN, 12–17 min-0% ACN. Fractions containing low molecular weight compounds (up to 8 min elution) indicated by the UV detector for the wavelength λ = 214 nm were discarded, while the fractions containing peptides (8–15 min elution) were collected, pooled, and dried under vacuum. The dried peptides were then dissolved in 0.1% formic acid solution, and their concentration was determined using the Direct-Detect system from Millipore^®^. The peptides in each sample were standardized to 5 µg and measured by LC-MS. which was then stored at −80 °C.

### 2.5. Quantitative and Qualitative Determination of Proteins-Proteomic Analysis by LC-MS

The ProteoMiner technology (Bio-Rad, Hercules, CA, USA) was used in order to decrease the amount of high-abundance proteins without immune-depletion, preventing the loss of proteins bound to high-abundance proteins, according to the manufacturer’s instructions.

Samples containing 5 µg of the plasma peptide was subjected to proteomic processing during which the peptides were separated on a C-18 nano-HPLC column using an acetonitrile gradient (5–35% for 180 min) in the presence of 0.1% formic acid. The chromatography column was coupled to a mass spectrometer operating in MS (peptide mass measurement) and MS/MS (peptide fragmentation) modes. Then the raw data was analyzed with an appropriate program using the Swiss-Prot database (taxonomy restricted to Homo sapiens). The cut-off point was <1% FDR. The list of peptides identified in all LC-MS runs was superimposed on the two-dimensional maps generated from the LC-MS profile data for the individual samples, based on the mass-to-charge (m/z) ratio, deviation from the predicted elution time, and the fit between the theoretical and observed mass ranges. Finally, a list of peptide ions was generated with their intensity for each sample. It was statistically analyzed using the Diffprot software and lists of statistically significant state-specific proteins were generated. Proteomic analysis was conducted in the Mass Spectrometry Laboratory at the Institute of Biochemistry and Biophysics of the Polish Academy of Sciences in Warsaw. All software used is accessible at http://proteom.ibb.waw.pl (accessed on 1 September 2018).

### 2.6. Statistical Analyses

The statistical analysis of demographic and biochemical parameters was performed with the use of the Statistica 10.0 StatSoft^®^ program. Data is expressed as mean ± SEM. The results at the level of *p* < 0.05 were considered statistically significant.

The differences between the means were assessed using the Student’s T-test or the Mann–Whitney U test depending on the distribution of the variables and the variety of variances. Post-hoc analysis was performed with Newman–Keuls tests. The Diffprot software [18] was used for the analysis of proteomic/peptidomic data, which allowed the generation of a list of significant levels of proteins in the plasma.

## 3. Results

### 3.1. Baseline Characteristics of Patients

Characteristics of the groups is presented in Table 1. Study and control groups were homogenous in terms of age, sex, and ethnicity (100% Caucasian). In subjects with ischemic stroke the increased white blood cells count (WBC) and the decreased mean platelet volume (MPV) has been reported when compared to the control group. Basic biochemical test also revealed the decreased level of potassium and high density lipoproteinin (HDL) in the stroke group when compared to controls.

### 3.2. LC-MS Results

Differential quantification of the plasma proteome revealed ten proteins with the significantly different concentrations between examined and control group. Alpha-1B-glycoprotein (A1B-GP) and the heat shock protein of the Hsp70 (Hsp70s) family differed significantly between the study group on the first day after stroke (group A) and the control group (group C). 

On the 7th day after the stroke, differences were found between the study group and the control group (group B vs. C) regarding complement factor B (Bf), apolipoprotein A-I (ApoA1), fetuin A, fibronectin (Fn), cytoplasmic tryptophanyl-tRNA synthetase (TrpRS) and ficolin-2.

The next two proteins (thymidine phosphorylase (TYMP) and beta-alanylhistidine dipeptidase (β-Ala His dipeptidase) differed significantly only when the combined group (group A + B), which characterizes all stroke patients regardless of the day of the test, was compared with the control group (group C) (Table 2).

## 4. Discussion

This is the first study to analyze the time-dependent changes in human plasma proteome during the acute phase of ischemic stroke using LC-MS. We identified ten proteins that could be biomarkers useful in the diagnosis of acute phase of ischemic stroke. Furthermore, observed proteome differences in blood during stroke may reflect a cascade of pathophysiological events related to the evolution of the disease and therefore help us to better understand the pathophysiology of cerebral ischemia.

### 4.1. Basic Biochemical Tests

The level of total cholesterol and its fractions was determined after the first 24 h. The decreased levels of LDL and CT correspond to the earlier findings that the levels of these parameters decreased on the first day after ischemic stroke. Significantly lowered HDL cholesterol level may be associated with an increased risk of cerebrovascular events. Previous data supports an inverse relationship between serum HDL levels and incidence of stroke [19]. Some prospective studies have demonstrated that higher HDL levels are associated with lower stroke risk in long-term observation [20,21], and an increase in of oxidative stress in HDL particles from patients after ischemic stroke is linked to decreased anti-oxidant enzyme paraoxonase 1 (PON-1) activity. Understanding the relationships between the pathophysiology and HDL-particle size distribution may better aid in risk assessment. Moreover, the question if subclasses of HDL differ in their anti-atherogenic functionality, the amount of apolipoprotein A-1, and ultimate efficiency in reverse cholesterol transport as well as the role of these phenomena in the risk for stroke, remain unanswered.

Increase in potassium concentration observed in many studies during acute ischemia of central nervous system (CNS) contributes to the damage of nervous tissue [22]. In our study, lower potassium level may be related to lower baseline values in patients more susceptible to stroke [23]. According to several reports, a diet with a high potassium intake reduces the risk of ischemic stroke and improves the prognosis after an ischemic event [24]. A recent meta-analysis confirms the inverse association between potassium intake and stroke risk, with potassium intake of 90 mmol (≈3500 mg)/day associated with the lowest risk of stroke [25] and several studies indicate protective effects of potassium against thrombus formation, atherosclerotic lesion progression, endothelial dysfunction, and free radical generation [26,27,28].

To date, elevated MPV has been a suggested risk factor for ischemic stroke, predicting the severity of the neurological deficit [29]. On the other hand, reduced MPV values were found in patients after intensive motor neurorehabilitation [30]. Interpretation of the reduced MPV values in the stroke population analyzed in this study is difficult since people with a severe course of the disease were not eligible for the study, and in this population, one would expect increased MPV values. According to some authors, elevated WBC plasma levels, most often associated with inflammatory processes, may be a predictor of ischemic stroke [31]. The increase in WBC in our study may be caused by an acute (infection) or chronic (atherosclerosis) inflammatory response, which in many cases is difficult to distinguish [32].

### 4.2. Proteomics Analysis

Differences were observed in the serum proteome of people with ischemic stroke compared to the control group, identifying two proteins: TYMP and β-Ala His dipeptidase. Levels of those parameters differed significantly only when the combined group all stroke patients, regardless of the day of the study, was compared with the control group. 

TYMP, also known as platelet-derived endothelial growth factor [33], catalyzes the phospholysis reaction of 2′-deoxytimidine to 2-deoxy-D-ribose-1-phosphate and thymine [34]. So far, its activity has been associated primarily with tumor growth. It promotes angiogenesis [35] and inhibits apoptosis of neoplastic cells [36]. Additionally, TYMP stimulates the growth and chemotaxis of endothelial cells [37] and regulates platelet activation and thrombosis [38]. The TYMP function in the brain is not fully understood. Under physiological conditions, astrocyte-derived TYMP along with vascular endothelial growth factor (VEGF) is responsible for the continuity of the blood-brain barrier, but during the inflammatory response, both proteins weaken this barrier function [39]. TYMP is also known as gliostatin [40]. It has a strong inhibitory effect on glial cells, maintaining homeostasis and protecting neurons in the central nervous system [41]. It has also been shown that TYMP expression increases in neurons after ischemia-reperfusion (IR) injury [42], but the role of this enzyme in the pathogenesis of ischemic brain damage is not well known. 

The activity of TYMP facilitating the formation of blood clots, disrupting homeostasis, and breaking the blood-brain barrier suggests that inhibition of this enzyme activity may lead to prevention of ischemic stroke. The use of a TYMP inhibitor has been shown to inhibit collagen and ADP-induced platelet aggregation, thereby inhibiting thrombosis without causing significant bleeding [43,44]. 

We postulate that TYMP-4 might become a therapeutic target [38,45] in human stroke, reducing the extent of ischemic penumbra in the acute phase of ischemic stroke. Since the TYMP-4 inhibitor molecule-tipiracil is already used in clinical practice (in the chemotherapy of colorectal cancer-as an adjuvant that inhibits the disintegration and increases the concentration of the chemotherapeutic-nucleoside analogue, trifluridine). We started the future direction of our study on the usefulness of TYMP-4 by tipiracil and other TYMP-4 inhibitors in reducing the extent of brain ischemia reperfusion injury in an animal model. In the present study, the increase in TYMP levels was observed in the acute phase of ischemic stroke. Therefore, it is important to consider the effect of TYMP inhibiting on outcome in humans after stroke. 

There are no reports of the role of β-Ala His dipeptidase in ischemic stroke. There is a single report about the role of this protein in patients with mucopolysaccharidosis (Anderson-Fabry disease) [46]. The accumulation of globotriaozylceramide in blood vessels and tissues leads to the development of cardiovascular complications such as heart attack, hypertrophic cardiomyopathy, and stroke. In the present study, elevated levels of β-Ala His dipeptidase were observed in the acute phase of ischemic stroke, which may reflect both its protective effect on brain tissue and the effects of ischemia during the acute phase of stroke. 

The dynamics of changes in the serum proteome of people with ischemic stroke in the determined time series was demonstrated, indicating the connection of certain proteins with the course of the disease. On the first day after stroke a1B-GP and Hsp70s differed significantly between the study group and the control group. 

A1B-GP has been described as a ligand for the cysteine rich secretory protein 3 (CRISP-3) [47], which is implicated in the tissue response to ischemia [48]. In the present study, decreased level of a1B-GP was observed in the acute phase of ischemic stroke. Due to the small amount of research, it can only be suspected that this protein is probably involved in both protective processes and ischemic damage of brain tissue. 

Much more in known about the involvement Hsp70s in the pathogenesis of stroke. In the present study, an increased level of the protein 8 (Hsp70-8, heat shock cognate 71 kDa protein) and the protein 2 (Hsp70-2, heat shock-related 70 kDa) was observed. It correlates with the results of other studies [49]. Hsp70-8 is a factor that can protect brain cells against ischemia cooperating with anti-apoptotic factors and reducing the area of cell death in the brain [50]. It has been shown that the expression of the HSPA8-ps1 gene is increased in the cerebral cortex affected by a stroke [51]. It is also worth noting that the expression of HSPA8 in the brain increases with age, which in the elderly may limit the negative effects of oxidative stress on nervous tissue [52]. The 15-deoxy-spergualin is among examined substances that modulate the function of Hsp70-8. Enhancing the activity of Hsp70-8 it could have a protective effect on brain tissue during of ischemic stroke [53].

The second protein, Hsp70-2, is mainly found in the testes and the brain. Elimination of the HSPA2 gene in mice leads to apoptosis of germ cells [54], suggesting that Hsp70-2 may play a similar anti-apoptotic role in the brain as well. Moreover, the synthesis of the Hsp70-2 protein is probably induced by ischemia [55] and therefore this protein could also be a therapeutic target in ischemic stroke. 

Only on the seventh day after the stroke differences were found regarding Bf, ApoA1, Fn, fetuin A, TrpRS and ficolin-2. 

The reduced concentration of Bf was observed in our work. Some studies demonstrated that the Bf is involved in the development of IR brain injury and neurological deficits [56,57] and the inhibition of this component might significantly reduce the post-stroke deficits [58]. Other reports suggest its participation in neuroprotection as an effect of natural brain remodeling after an ischemic event [59,60]. It also may represent a post-stroke immunosuppression [61]. A study on mice suggests that administration of an anti-factor B antibodies may reduce necrotic damage to brain cells, but without improving the neurological deficit [62].

In line with other reports [63], in our study a decreased concentration of Apo A1 was observed on the 7th day after the stroke. However, some authors suggested that the usefulness of ApoA1 appears to be of little use in the early diagnosis of stroke as its concentrations do not change significantly in the first 28 days following an ischemic event [64]. Indicating the presence of atherosclerosis, ApoA1 is also a prognostic parameter of an increased risk of neurovascular events [65]. Increased levels of anti-ApoA1 IgG antibodies have been shown to be associated with higher risk of major cardiovascular events including stroke [66]. There are still no data available to conclusively explain whether the decreased ApoA1 level belongs to the pathophysiology of the acute phase of stroke or is only associated with an increased risk of stroke in this group. 

The differences in the Fn level were also found in our work on 7th day after the stroke. Based on available data Fn is considered a marker of the blood-brain barrier damage during stroke [67]. It is involved in the formation of a blood clot and inflammation [68]. In some reports the concentration of this protein in plasma appears to be dependent on the duration of ischemia, peaking between 6th-8th hours [69]. A relationship was found between the concentration of Fn and the occurrence of hemorrhagic stroke [70] and the risk of hemorrhagic transformation after thrombolytic management of ischemic stroke [71]. 

Studies in rats have shown that fetuin A has an anti-inflammatory effect, resulting in tissue protection from cerebral ischemic injury [72]. In the present study, increased level of fetuin A was observed which correlates with the results of other studies that have shown a relationship between fetuin A concentration and the severity of ischemic stroke. A change in the concentration of this parameter may indicate prognosis and subsequent recovery [73]. 

Under hypoxic conditions, the expression of TrpRS is reduced, which was demonstrated, among others, by on the cells of the pancreatic cells [74]. Our findings showed that the level of this protein decreases in the acute phase of ischemic stroke. It is commonly observed that at the same conditions there is an increased expression of another form of this enzyme, mini-TrpRS, which impairs myocardial perfusion after infarction in rat [75] and in monkeys [76]. As an antagonist of ocular angiogenesis, it may also have a beneficial effect in inhibiting pathological neovascularization [77]. The role of TrpRS and mini-TrpRS in the pathogenesis of ischemic stroke requires further research. 

On the 7th day after the onset of ischemic stroke symptoms, our study also showed an increased concentration of ficolin-2. Similar results were presented in previous reports. Zangari et al. [78] observed decreased plasma levels of ficolin-2 after 6 h of symptom duration, which increased slightly between the 3rd and 5th days and one month after stroke. In another study, Füst et al. [79] reported decreased levels of ficolin-2 on days 1 and 3 after stroke. Such results suggest that changes in ficolin-2 concentration would become a useful marker of the acute phase of ischemic stroke. 

## 5. Conclusions

This is the first dynamic LC-MS study performed on a clinical model which differentiates serum proteome of patients in acute phase of ischemic stroke in time series and compares to control group. Our study showed 10 proteins with significantly different concentrations between the groups. Listed proteins should be considered as risk factors, markers of ischemic stroke or potential therapeutic targets. Our understanding of their role in ischemic stroke requires further research. Similarly, there is a need for their validation as human biomarkers of ischemic stroke. Nevertheless, in the future, they could be used as biomarkers to better diagnose ischemic stroke and develop new therapeutic strategies.

## Figures and Tables

**Table 1:** Demographic and biochemical characteristics selected groups, with particular emphasis on cardiovascular risk factors.

		Stroke (Mean ± SEM) *n* = 31			Control (Mean± SEM) *n* = 28			*p*
Women		13 (42%)			14 (50%)			0.38
Age	[y]	62.68	±	9.35	62.00	±	11.40	0.80
Hemoglobin	[g/dL]	14.39	±	1.63	13.78	±	1.63	0.16
Hematocrit	[%]	42.65	±	4.45	40.93	±	4.81	0.16
RBC	[m/µL]	4.79	±	0.59	4.68	±	0.58	0.50
WBC	[k/µL]	9.16	±	3.48	7.05	±	2.55	0.01 *
PLT	[k/µL]	254	±	195	244	±	46	0.14
MPV	[fl]	9.38	±	1.26	10.90	±	0.83	0.00 *
hsCRP	[mg/L]	5.55	±	6.03	5.52	±	5.96	0.99
Potassium	[mmol/L]	3.85	±	0.34	4.15	±	0.42	0.00 *
Sodium	[mmol/L]	139	±	2.50	139.38	±	5.23	0.06
Glucose	[mg/dL]	130	±	50	108	±	51	0.12
Urea	[mg/dL]	33.7	±	12.7	29.5	±	13.1	0.22
Creatinine	[mg/dL]	0.93	±	0.27	0.89	±	0.27	0.57
AST	[IU/L]	19.00	±	7.06	23.50	±	12.00	0.22
ALT	[IU/L]	21.63	±	9.79	26.65	±	15.75	0.31
Total bilirubin	[mg/dL]	0.54	±	0.07	0.72	±	0.29	0.30
TCh	[mg/dL]	184.27	±	48.20	210.95	±	52.34	0.06
HDL	[mg/dL]	49.20	±	13.60	58.36	±	15.87	0.03 *
LDL	[mg/dL]	108.83	±	41.47	126.22	±	47.50	0.16
TG	[mg/dL]	137.23	±	92.44	144.48	±	84.09	0.77
TSH	[µIU/L]	3.03	±	3.56	1.47	±	0.85	0.06
APTT	[s]	27.66	±	3.58	28.61	±	5.22	0.45
PT/INR		0.98	±	0.10	0.99	±	0.05	0.18

*—*p* statistically significant factor (*p* < 0.05); *p*, test probability; SEM, standard error of the arithmetic mean.

**Table 2:** Quantitative differences of found proteins.

Protein Name	Comparison		q Value	Ratio	Fold Change	Peptides
Complement factor B	A vs. C	p1	0.13460	0.51	1.97	69
		p2	0.37151	0.49	2.04	69
	**B vs. C**	**p1**	0.00032 *	**0.52**	**1.92**	**70**
		**p2**	0.00015 *	**0.53**	**1.87**	**70**
	**A + B vs. C**	**p1**	0.03326 *	**0.56**	**1.80**	**70**
		p2	0.15461	0.59	1.69	69
Apolipoprotein A-I	A vs. C	p1	1.00000	0.69	1.45	405
		p2	1.00000	0.75	1.34	405
	**B vs. C**	**p1**	0.00021 *	**0.63**	**1.59**	**405**
		**p2**	0.00034 *	**0.65**	**1.53**	**405**
	A + B vs. C	p1	1.00000	0.64	1.57	407
		p2	1.00000	0.62	1.61	405
Fibronectin	A vs. C	p1	0.78238	1.57	1.57	12
		p2	0.68263	1.43	1.43	12
	B vs. C	p1	0.08008	1.95	1.95	12
		**p2**	0.03980 *	**2.15**	**2.15**	**12**
	A + B vs. C	p1	0.24051	1.43	1.43	12
		p2	0.77102	1.40	1.40	12
Alpha-2-HS-glycoprotein	A vs. C	p1	0.69703	1.84	1.84	126
		p2	0.72959	1.66	1.66	126
	B vs. C	**p1**	0.01408 *	**1.62**	**1.62**	**126**
		**p2**	0.00015 *	**1.63**	**1.63**	**126**
	A + B vs. C	p1	0.29914	1.39	1.39	126
		p2	0.87082	1.53	1.53	126
Alpha-1B-glycoprotein	**A vs. C**	**p1**	0.00290 *	**0.54**	**1.87**	**55**
		**p2**	0.04753 *	**0.52**	**1.91**	**54**
	B vs. C	p1	1.00000	0.56	1.78	55
		p2	0.90000	0.57	1.74	53
	A + B vs. C	p1	0.05477	0.46	2.19	55
		**p2**	0.00146 *	**0.40**	**2.53**	**54**
Heat shock protein Hsp70 family †	A vs. C	**p1**	0.00663 *	**2.19**	**2.19**	**1**
	B vs. C	p1	1.00000	2.11	2.11	1
	A + B vs. C	p1	1.00000	1.51	1.51	1
Thymidine phosphorylase	A vs. C	p1	0.77044	0.23	4.35	1
	B vs. C	p1	0.57787	0.13	7.55	1
	**A + B vs. C**	**p1**	0.00425 *	**0.12**	**8.05**	**1**
Tryptophan--tRNAligase, cytoplasmic	A vs. C	p1	0.85112	0.44	2.29	1
	**B vs. C**	**p1**	0.00163 *	**0.18**	**5.55**	**1**
	**A + B vs. C**	**p1**	0.00230 *	**0.25**	**3.96**	**1**
Ficolin-2	A vs. C	p1	0.67919	2.77	2.77	5
		p2	0.68670	2.11	2.11	5
	**B vs. C**	**p1**	0.01077 *	**6.53**	**6.53**	**5**
		**p2**	0.04484 *	**5.18**	**5.18**	**5**
	A + B vs. C	p1	0.29495	1.67	1.67	5
		p2	0.34767	2.55	2.55	5
Beta-Ala-His dipeptidase	A vs. C	p1	0.14225	6.47	6.47	7
		p2	0.35263	6.10	6.10	7
	B vs. C	p1	0.08966	2.58	2.58	7
		p1	0.06369	3.13	3.13	7
	**A + B vs. C**	**p1**	0.04009 *	**1.49**	**1.49**	**7**
		p2	0.13244	1.16	1.16	7

*p* (n)-The minimum number of peptides at which the protein is included in the analysis. A, B,-examined groups, C- control group. *—statistically significant q factor (q < 0.05); q, test probability; A-blood sampling on day 1 of hospitalization; B-blood sampling on the 7th day of hospitalization; C-blood sampling from the control group; A vs. C-difference at the beginning of the observation; B vs. C-difference after seven days of observation; A + B vs. C-difference present between the combined samples of the study group compared to the control group; *p* (n)-the minimum number of peptides in which the protein is included in the analysis; †—The set of peptides assessed corresponds to the sequence of protein 2 and protein 8 from the Hsp70 family.

## Data Availability

The data presented in this study are available on request from the corresponding author.
